# Effects of Selenium-Enriched Yeast on Laying Performance, Egg Quality, Selenium Deposition, and Serum Biochemical Parameters in Laying Hens

**DOI:** 10.3390/ani16162506

**Published:** 2026-08-12

**Authors:** Qiqi Chen, Xiaobin Feng, Yang Song, Zhiwei Zhang, Yanli Jia, Kangzhuang Zhang, Xinyu Xie, Wei Dong, Zhenzhen Liu

**Affiliations:** 1 Institute of Food Science Technology Nutrition and Health (Cangzhou), Chinese Academy of Agricultural Sciences, Cangzhou 061000, China; 17852636857@163.com (Q.C.); fengxiaobin@126.com (X.F.); m15930277760@163.com (Y.S.); 13131751293@163.com (Z.Z.); jiayanli1988@126.com (Y.J.); 13393277347@163.com (K.Z.); m18845638861@163.com (X.X.); 2 Cangzhou Academy of Agriculture and Forestry Sciences, Cangzhou 061001, China

**Keywords:** Hy-Line Brown laying hens, selenium-enriched yeast, selenium deposition, serum biochemical parameters

## Abstract

Selenium (Se) is essential for livestock, and selenium-enriched yeast (SY) is widely used to produce Se-enriched eggs. However, the optimal dosage for late-peak laying hens remains unclear. This 8-week trial evaluated graded SY (0, 0.1, 0.3, and 0.5 mg/kg Se) in 28-week-old Hy-Line Brown laying hens to explore the comprehensive effects. SY did not affect laying performance or egg quality (*p* > 0.05), whereas egg Se content increased dose-dependently from 0.211 to 0.521 mg/kg (*p* < 0.001). SY enhanced antioxidant capacity, with glutathione peroxidase and catalase activities rising from 90.67 and 7.91 U/mL to 493.33 and 21.43 U/mL (*p* < 0.001), and malondialdehyde decreasing from 0.46 to 0.13 nmol/L (*p* < 0.001). Serum lipid parameters were markedly improved, with total cholesterol and triglycerides declining from 8.82 and 17.60 mmol/L to 2.86 and 13.73 mmol/L (*p* < 0.001). Humoral immunity was also positively modulated, as immunoglobulin G and A increased from 401.68 and 73.06 μg/mL to 756.30 and 115.80 μg/mL (*p* < 0.001). Integrating egg enrichment efficiency, laying hen physiological health, and economic benefits, 0.3 mg/kg Se was identified as optimal, achieving 0.358 mg/kg egg Se. This study provides a novel paradigm for standardized and cost-effective commercial production of Se-enriched eggs.

## Simple Summary

Selenium is essential for humans, and selenium-enriched eggs are an easy way to supplement selenium. However, farmers lack clear standards for selenium supplementation in feed. An 8-week feeding trial was conducted using late peak Hy-Line Brown laying hens. Three supplemental selenium levels derived from selenium-enriched yeast were set in the experiment, namely low (0.1 mg/kg), medium (0.3 mg/kg), and high (0.5 mg/kg). We tested laying performance, egg quality, selenium accumulation in eggs and serum, and serum biochemical parameters to assess comprehensive effects on hens. Results showed selenium supplementation did not change laying performance or egg quality, yet moderate selenium boosted hens’ antioxidant capacity, immunity, and lipid metabolism. Low selenium doses cannot meet the standard for selenium-enriched eggs. Excess selenium pushes egg selenium above the standard limit, raising economic benefits without additional hen health benefits. Balancing egg selenium enrichment efficiency, laying hen physiological health, and economic benefits, 0.3 mg/kg selenium from selenium yeast was identified as the optimal dose, producing qualified eggs with 0.358 mg/kg selenium. This study offers cost-effective feeding guidance for farms to produce standard selenium-enriched eggs.

## 1. Introduction

Selenium (Se) is an indispensable trace element for humans and animals, acting as a catalytic cofactor for over 25 selenoproteins and multiple functional enzymes [1,2]. By being integrated into glutathione peroxidases (GSH-Px), thioredoxin reductases, and iodothyronine deiodinases, Se participates in the regulation of redox homeostasis, immune response, and thyroid hormone metabolism, while protecting the liver and tissue integrity and relieving oxidative stress [3,4,5]. In poultry, Se deficiency can lead to exudative diathesis, pancreatic degeneration, declined egg production, deteriorated egg quality, suppressed immunity, and elevated mortality [6,7,8]. On the contrary, appropriate Se supplementation is capable of improving growth performance, optimizing reproductive function, and reinforcing the antioxidant defense system [9,10,11].

Sodium selenite is the most commonly used inorganic Se supplement in poultry diets due to its low cost. However, it has poor bioavailability and can pose toxic risks if overdosed [12,13]. In contrast, organic Se, particularly in the form of selenium-enriched yeast (SY), has gained significant attention in research for its superior bioavailability, greater capacity to accumulate Se in tissues, and lower toxicity risk [14,15,16,17]. Previous studies have shown that SY is much more effective at depositing Se in table eggs compared to inorganic sources [18]. The high content of selenomethionine in SY contributes to its excellent absorption and effectiveness in enriching eggs with Se [19,20,21].

Se-enriched eggs serve as an excellent dietary source to help alleviate Se deficiency in human populations. Each enriched egg contains approximately 20–25 μg of Se, which accounts for nearly 30% of the daily Se requirement for adults [22,23]. According to the Chinese industry standard GH/T 1135-2017 [24], the Se content of qualified Se-enriched eggs is regulated within 0.15–0.50 mg/kg, which guarantees nutritional value and food safety for consumers. Against this standardized threshold, identifying proper SY supplemental dosages for laying hens carries great practical value for large-scale egg production.

Substantial studies have systematically explored the biological effects of SY supplementation in laying hen breeding. Appropriate SY supplementation has been proven to ameliorate egg quality and boost antioxidant capacity via activating the hepatic nuclear factor erythroid 2-related factor 2 (Nrf2) signaling pathway and elevating the activities of GSH-Px, superoxide dismutase (SOD), and catalase (CAT) [2,3,25,26]. Li et al. [14] reported that 0.15 mg/kg dietary organic Se markedly increased serum immunoglobulin G (IgG) and immunoglobulin A (IgA) levels in laying hens. Wang et al. [25] illustrated that SY supplementation at 0.3–0.9 mg/kg Se improved egg quality and hepatic antioxidant activity in a dose-dependent pattern. For senescent 76-week-old laying hens, it was verified that 0.30 mg/kg Se from SY could enhance egg production by regulating ovarian function and intestinal microbiota [7]. A meta-analysis covering 81 published papers further confirmed the overall superiority of organic Se over inorganic Se in egg Se deposition and antioxidant regulation of layers [27].

Despite considerable progress in previous research, several critical gaps still impede the standardized application of SY in laying hen production. First, existing evidence has been derived predominantly from aged hens or heat-stress models [6,14,17], and it remains unclear whether these findings can be extrapolated to healthy hens during the late stage of peak production (28 to 36 weeks of age). Second, although it is well established that egg Se content increases with rising dietary Se levels [9,17,19], no quantitative predictive equation linking dietary Se supplementation to egg Se content has yet been established for Hy-Line Brown laying hens. As a result, farms cannot estimate whether their table eggs will comply with the national standard (0.15–0.50 mg/kg, GH/T 1135-2017) [24]. Third, while some studies have examined various biochemical parameters in laying hens, most have relied on single-variable phenotypic analyses; few have employed multivariate approaches to integrate physiological responses with graded Se supplementation for optimal dosages [17,25,28,29]. In summary, the optimal SY dosage for late-peak laying hens that simultaneously balances egg Se enrichment efficiency, laying hen physiological health, and economic benefits remains to be determined. These gaps directly hinder the precision and standardization of Se-enriched egg production [29]. Most importantly, for laying hens at the late peak laying stage (28–36 weeks), the optimal SY dosage that comprehensively balances egg Se enrichment, animal physiological health, and production economy has not yet been determined, which hinders the precise and safe production of qualified Se-enriched eggs.

To address the aforementioned gaps, this 8-week trial was performed on 28–36-week-old Hy-Line Brown laying hens fed diets supplemented with graded SY-derived Se (0, 0.1, 0.3, and 0.5 mg/kg). Laying performance, egg quality, and egg or serum Se content were systematically measured, and a quantitative dose–response equation linking dietary Se level to Se content was established. In parallel, a panel of 19 serum biochemical parameters was assessed to reflect antioxidant status, immune function, lipid metabolism, and hepatic and renal function. Multivariate statistical analyses, including principal component analysis (PCA), hierarchical clustering, and radar charting, were then performed to comprehensively characterize the physiological status of hens across different supplementation levels. Collectively, this study sought to determine the optimal SY dosage that best balances egg Se enrichment efficiency, laying hen physiological health, and economic benefits, thereby providing a practical basis for the standardized production of Se-enriched eggs in commercial layer farms.

## 2. Materials and Methods

### 2.1. Animal Ethics Statement

All experimental procedures were approved by the Animal Ethics Committee of Hebei University (Approval No. HBUM2025079). All animal manipulations in this study were performed in accordance with the ARRIVE 2.0 guidelines [30].

### 2.2. Experimental Animals, Diets, and Feeding Management

A total of 60 healthy 28-week-old Hy-Line Brown laying hens with similar body weights were obtained from Aosheng Family Farm (Cangzhou, China). The hens were randomly assigned to four treatment groups, with 15 hens per group. Hens in the negative control group (NC) were fed a Se-free basal diet, while birds in the three treatment groups (SY1, SY2, and SY3) received the same basal diet supplemented with SY to achieve a dietary Se content of 0.1, 0.3, and 0.5 mg/kg, respectively. The applied SY product contained 2000 mg/kg Se (Qinshengyuan Biotechnology Co., Ltd., Xi’an, China). According to the target Se levels, the corresponding supplemental SY dosages were 0.05, 0.15, and 0.25 g/kg diet for the SY1, SY2, and SY3 groups, respectively. All basal diets were formulated in compliance with the Chinese Feeding Standard of Chicken (NY/T 33-2004) [31], and the composition and nutritional levels of the basal diet are presented in Table 1.

15 hens per treatment group housed in one cage (130 cm × 60 cm × 70 cm). Each cage was evenly divided into three independent compartments by wire mesh baffles, and each compartment measured approximately 43 cm × 60 cm × 70 cm. Five individually tagged laying hens were raised in each compartment, which was defined as one intra-cage sub-replicate. The usable floor area of each compartment was 2580 cm^2^, providing 516 cm^2^ of effective activity space per hen. This value slightly exceeds the cage space requirement of 420–500 cm^2^ per laying hen specified in the agricultural industry standard Specifications for Construction of Standardized Layer Farms (NY/T 4828-2025) [32]. We intentionally adopted a slightly higher stocking density to eliminate crowding-induced oxidative stress. The entire feeding trial lasted for 8 weeks, covering the laying period from 28 to 36 weeks of age. During the experimental period, the chicken house was maintained under well-ventilated, dry, and hygienic conditions. The ambient temperature was kept at 18–22 °C, relative humidity at 50–60%, and the photoperiod was set as 16 h of light and 8 h of dark. No clinical health abnormalities were observed in any hens throughout the trial.

### 2.3. Sample Collection and Measurements

#### 2.3.1. Laying Performance

During the trial, daily feed consumption, total egg number, and individual egg weight were recorded for each intra-cage sub-replicate to calculate laying performance parameters, including average daily feed intake (ADFI), egg production rate (EPR), average egg weight (AEW), and feed-to-egg ratio (F/E).

ADFI (g) was calculated as total feed intake divided by the product of hen number and experimental days. EPR (%) was defined as the percentage of total egg number relative to the total number of hen-days. AEW (g) was determined by dividing total egg weight by total egg count. F/E was calculated as the ratio of total feed consumption to total egg weight.

#### 2.3.2. Egg Quality

On the 28th and 56th days of the trial, all fresh eggs were randomly collected from each intra-cage sub-replicate and stored at 4 °C before immediate egg quality determination. The longitudinal and transverse diameters of each egg were measured using a vernier caliper, and the egg shape index (ESI) was calculated as the ratio of transverse diameter to longitudinal diameter.

Egg and eggshell weight were measured with an electronic balance (ES-E220A, Deante Sensing Technology Co., Ltd., Tianjin, China), and the yolk was separated and weighed to calculate the eggshell ratio (ESR) and yolk ratio (YR). An egg quality analyzer (EA-01, Orka Technology Ltd., Ramat Hasharon, Israel) was used to determine albumen height (AH), Haugh unit (HU), and yolk color (YC), while eggshell thickness (EST) was detected using a spiral micrometer.

#### 2.3.3. Serum Biochemical Parameters

Feed was removed on the evening of day 55 for overnight fasting. In the early morning of the next day, 5 mL of blood was collected from the wing vein of each laying hen. After standing for 30 min at room temperature, blood samples were centrifuged at 3000 rpm for 15 min at 4 °C using a refrigerated centrifuge (CHT210R, Xiangyi Centrifuge Instrument Co., Ltd., Changsha, China) to separate serum. For each treatment group, the 15 laying hens were divided into 3 intra-cage sub-replicates (5 hens per sub-replicate). Serum collected from the 5 hens within the same intra-cage sub-replicate was pooled in equal volumes to prepare one composite serum sample for the detection of all serum biochemical parameters. The harvested composite serum samples were stored at −80 °C until subsequent biochemical analysis.

A series of serum biochemical parameters, including total antioxidant capacity (T-AOC), SOD, GSH-Px, CAT, and malondialdehyde (MDA), were measured using commercial assay kits purchased from Nanjing Jiancheng Bioengineering Institute (Nanjing, China). Liver and renal function parameters, including alanine transaminase (ALT), aspartate transaminase (AST), creatinine (CRE), urea (URE), and blood Urea nitrogen (BUN), were detected with commercial kits from Beijing Leadman Biochemistry Co., Ltd. (Beijing, China). Serum protein and lipid metabolism parameters, including total protein (TP), albumin (ALB), total cholesterol (TC), triglyceride (TG), high-density lipoprotein cholesterol (HDL-c), and low-density lipoprotein cholesterol (LDL-c), were also determined using kits from the same supplier. IgG, IgA, and immunoglobulin M (IgM) were quantified by enzyme-linked immunosorbent assay (ELISA) using corresponding ELISA kits supplied by Jingmei Biotechnology Co., Ltd. (Yancheng, China). All experimental procedures were performed in accordance with the standard protocols provided by the kit manufacturers. The instruments utilized for measuring the aforementioned serum biochemical parameters included an ultraviolet spectrophotometer (UV-1900I, Shimadzu Corporation, Kyoto, Japan), a fully automatic biochemical analyzer (Hitachi 3100, Hitachi High-Tech Corporation, Tokyo, Japan), and a microplate reader (Multiskan FC, Thermo Fisher Scientific Instruments Co., Ltd., Shanghai, China).

#### 2.3.4. Se Deposition in Eggs and Serum

Fresh eggs were collected from each sub-replicates from the 55 to 56 days of the trial. Whole egg contents from the same sub-replicate were fully mixed to form a composite sample for Se detection. Serum samples were prepared following the procedures described in Section 2.3.3. The Se contents in composite egg and serum samples were determined by hydride generation atomic fluorescence spectrometry, which is the first method specified in the National Food Safety Standard for Determination of Se in Foods (GB 5009.93-2017) [33]. Measurements were performed by an atomic fluorescence spectrophotometer (SK-2003A-LC, Jinsuokun Technology Development Co., Ltd., Beijing, China).

### 2.4. Statistical Analysis

All raw data were preliminarily sorted using Microsoft Excel 2019. One-way ANOVA of variance was performed using SPSS 19.0, and Duncan’s multiple range test was adopted for multiple comparisons among groups. All results are presented as mean ± standard deviation, with 3 intra-cage sub-replicates per treatment (5 laying hens per sub-replicate, 15 hens total per group). A difference of *p* < 0.05 was considered statistically significant. After data normalization, all multivariate visual analyses, including Pearson correlation heatmaps, Euclidean hierarchical clustering, PCA, and radar charts, were performed using Origin 2024. For hierarchical clustering, Euclidean distance was adopted as the distance metric, and Ward’s linkage was selected as the agglomerative linkage method for sample grouping. The sample grouping patterns derived from hierarchical clustering were qualitatively cross-referenced against the sample separation observed in PCA score plots to judge the rationality of cluster partitions.

## 3. Results

### 3.1. Laying Performance

The laying performance of hens fed diets supplemented with different levels of SY is summarized in Table 2. During the 8-week trial, dietary SY supplementation at 0.1, 0.3, and 0.5 mg/kg Se exerted no significant effects on AEW, EPR, F/E, and ADFI across all treatment groups (*p* > 0.05).

### 3.2. Egg Quality

Egg quality parameters measured at 4 and 8 weeks are listed in Table 3. No significant differences in EST, ESR, YR, YC, AH, and HU were observed between NC and all SY-supplemented groups at either sampling time point (*p* > 0.05). At 8 weeks of feeding, the ESI in the SY2 group was significantly lower than that in the NC group (*p* = 0.013), while no obvious differences were observed among the other treatments (*p* > 0.05).

### 3.3. Se Deposition in Eggs and Serum

**Figure 1:**
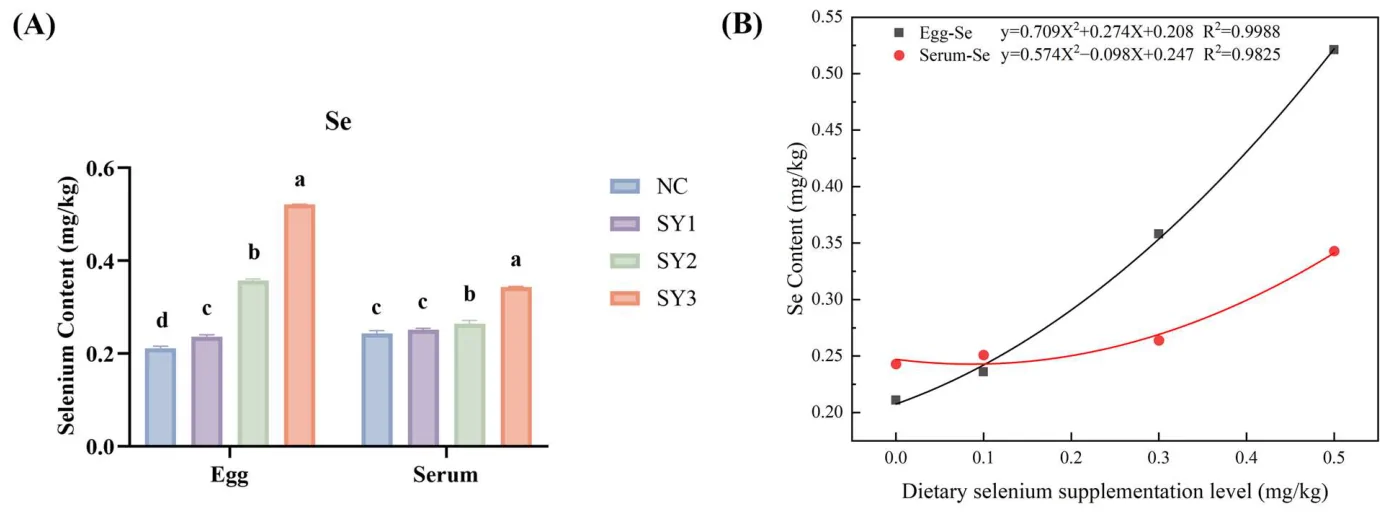
Effects of graded dietary SY supplementation on Se contents in eggs and serum. (**A**) Results of Se Contents in Eggs and Serum. (**B**) Quadratic fitting curves between dietary Se level and Se contents in eggs and serum. NC, negative control; SY1, 0.1 mg/kg; SY2, 0.3 mg/kg; SY3, 0.5 mg/kg. The bars labeled a–d with no common superscript differ significantly (*p* < 0.05). *n* = 3 intra-cage sub-replicates per treatment (5 laying hens per sub-replicate, 15 hens total per group).

Figure 1A shows egg Se content (mg/kg, fresh weight) and serum Se content (mg/kg, frozen weight) in all groups. Egg Se content differed significantly among treatments (*p* < 0.001). NC had the lowest egg Se content at 0.211 ± 0.005 mg/kg, whereas SY3 had the highest level at 0.521 ± 0.001 mg/kg, which was significantly greater than the other three groups (*p* < 0.001). Compared with the Se content of serum (0.243 ± 0.006 mg/kg), the Se content in the serum of SY1 showed no significant difference from it (*p* > 0.05). SY3 had the highest serum Se content, at 0.343 ± 0.002 mg/kg, which was significantly higher than other groups (*p* < 0.001).

The quadratic fitting curves of dietary Se supplementation levels versus Se concentration in eggs and serum were displayed in Figure 1B. The fitting equations were established as y = 0.709X^2^ + 0.274X + 0.208 (R^2^ = 0.9988) and y = 0.574X^2^ − 0.098X + 0.247 (R^2^ = 0.9825). Both R^2^ values were close to 1, revealing a significant quadratic dose-dependent accumulation pattern of Se in egg and serum.

### 3.4. Serum Biochemical Parameters

#### 3.4.1. Antioxidant Capacity

**Figure 2:**
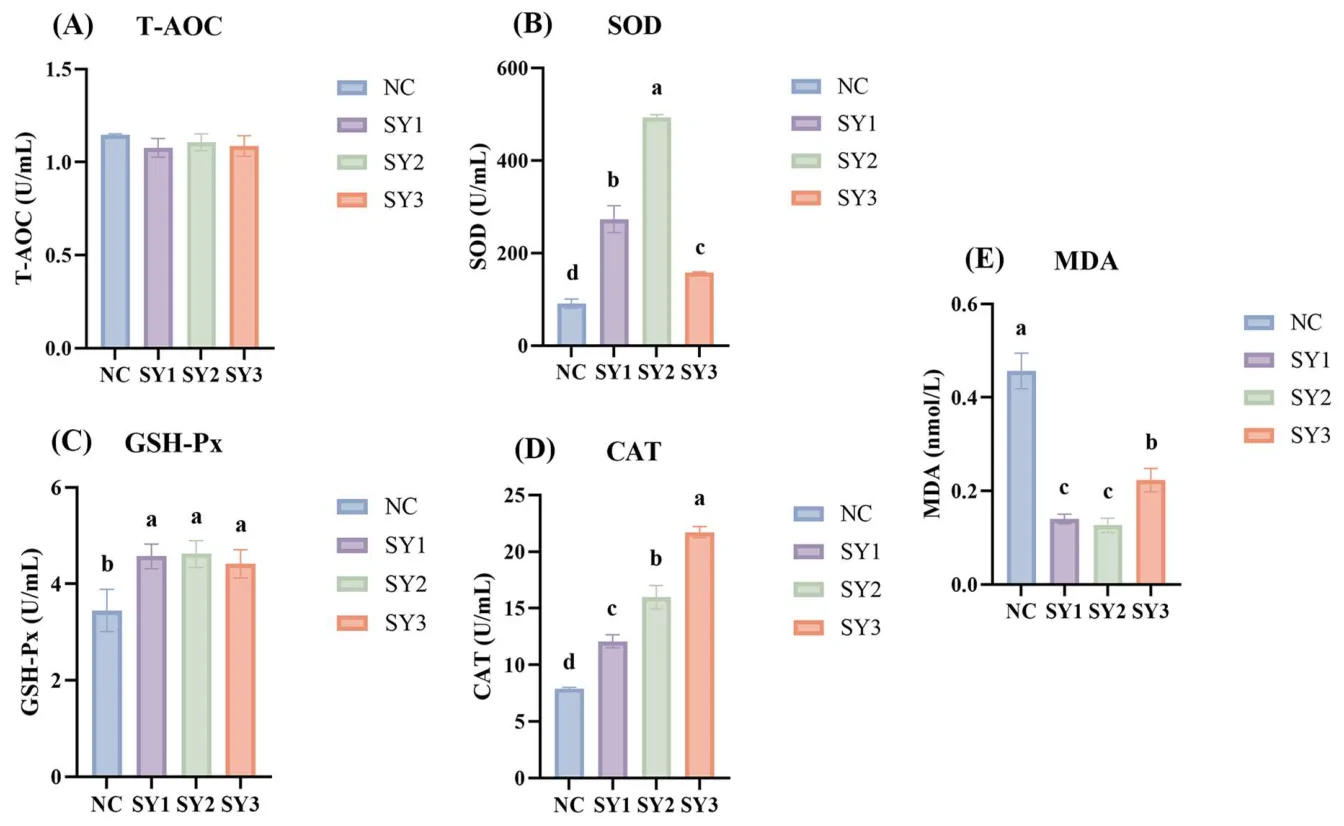
Effects of graded dietary SY supplementation on antioxidant capacity of laying hens. NC, negative control; SY1, 0.1 mg/kg; SY2, 0.3 mg/kg; SY3, 0.5 mg/kg. (**A**) T-AOC, total antioxidant capacity. (**B**) SOD, superoxide dismutase. (**C**) GSH-Px, glutathione peroxidase. (**D**) CAT, catalase. (**E**) MDA, malondialdehyde. The bars labeled a–d with no common superscript differ significantly (*p* < 0.05). *n* = 3 intra-cage sub-replicates per treatment (5 laying hens per sub-replicate, 15 hens total per group).

Antioxidant parameters are shown in Figure 2. T-AOC did not differ significantly among all groups (*p* > 0.05). SOD activity increased first and then decreased as dietary SY dosage rose. All SY groups had markedly higher SOD activity than NC (90.67 ± 10.12 U/mL), and SY2 exhibited the peak SOD activity at 493.33 ± 10.12 U/mL (*p* < 0.001). GSH-Px activity in the three SY groups was significantly higher than that in NC (3.45 ± 0.44 U/mL) (*p* = 0.003, *p* = 0.002, and *p* = 0.007, respectively), whereas no significant difference was found among SY1, SY2, and SY3 (*p* > 0.05). The highest GSH-Px activity (4.62 ± 0.28 U/mL) was recorded in SY2. CAT activity increased linearly from 7.91 ± 0.09 U/mL (NC) with increasing SY supplementation. SY3 had the maximum CAT activity at 21.43 ± 0.49 U/mL, which was significantly different from other groups (*p* < 0.001). MDA concentration in all SY groups was significantly lower than that in NC (0.46 ± 0.04 nmol/L) (*p* < 0.001), and the lowest MDA level (0.13 ± 0.02 nmol/L) was detected in SY2.

#### 3.4.2. Liver and Kidney Function

**Figure 3:**
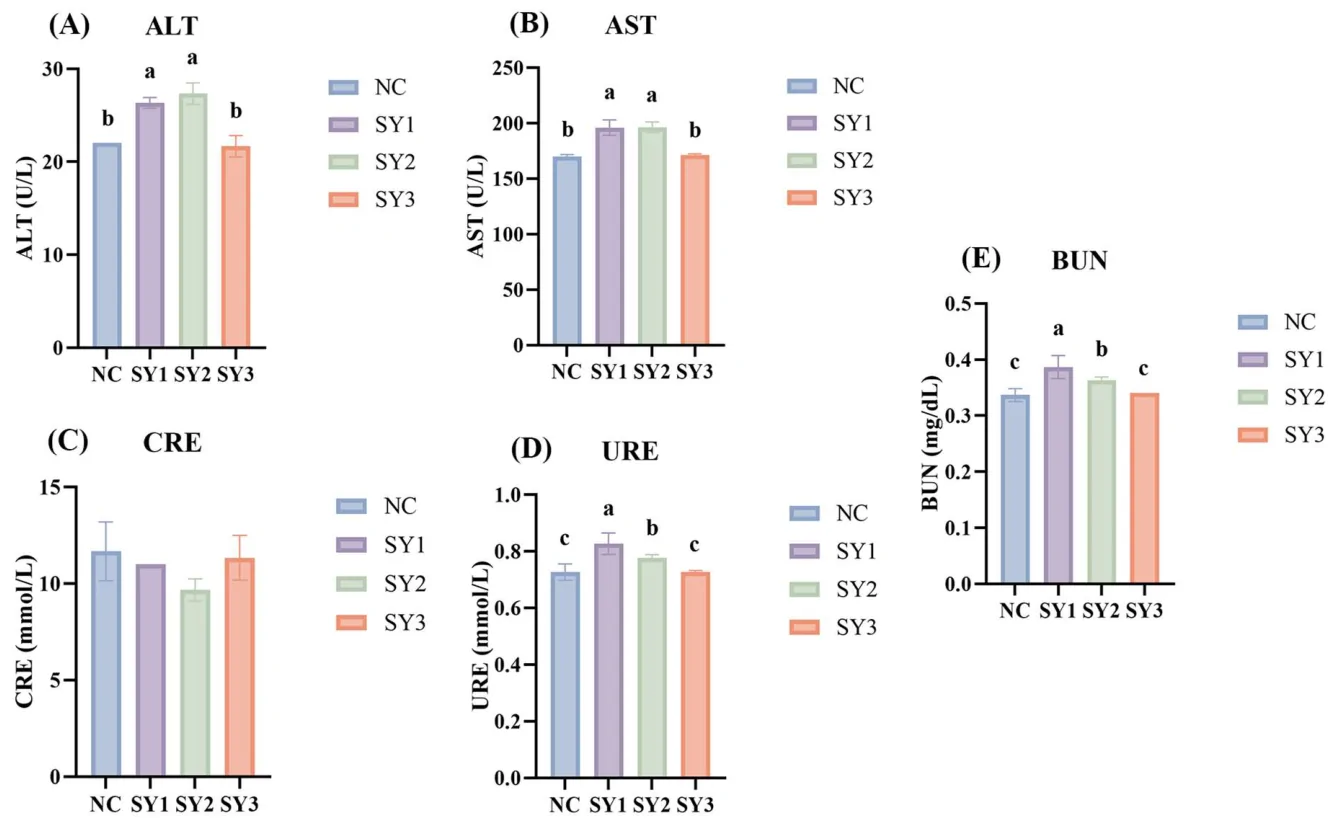
Effects of graded dietary SY supplementation on serum liver and kidney function of laying hens. NC, negative control; SY1, 0.1 mg/kg; SY2, 0.3 mg/kg; SY3, 0.5 mg/kg. (**A**) ALT, alanine transaminase. (**B**) AST, aspartate transaminase. (**C**) CRE, creatinine. (**D**) URE, Urea. (**E**) BUN, blood Urea nitrogen. The bars labeled a–c with no common superscript differ significantly (*p* < 0.05). *n* = 3 intra-cage sub-replicates per treatment (5 laying hens per sub-replicate, 15 hens total per group).

Figure 3 displays serum parameters related to hepatic and renal functions in each group. ALT and AST activities in SY1 and SY2 were significantly higher than those in NC (22.00 ± 0.00, 170 ± 1.73 U/L, respectively) (*p* < 0.001), and no significant difference existed between SY1 and SY2 (*p* > 0.05). SY2 had the highest ALT (27.33 ± 1.15 U/L) and AST (196.33 ± 4.73 U/L) activities. ALT and AST levels in SY3 were comparable to NC (*p* > 0.05). CRE concentration remained stable in all groups (*p* > 0.05). URE concentrations in SY1 and SY2 were significantly elevated relative to NC (*p* = 0.001 and *p* = 0.038, respectively), with no difference between the two groups (*p* > 0.05). BUN concentrations in the SY1 and SY2 groups were also significantly higher than those in the NC group (*p* = 0.01 and *p* = 0.029, respectively). SY1 presented the highest URE (0.83 ± 0.04 mmol/L) and BUN (0.39 ± 0.02 mmol/L) levels. URE and BUN concentrations in SY3 showed no significant difference from NC (*p* > 0.05).

#### 3.4.3. Protein Metabolism

**Figure 4:**
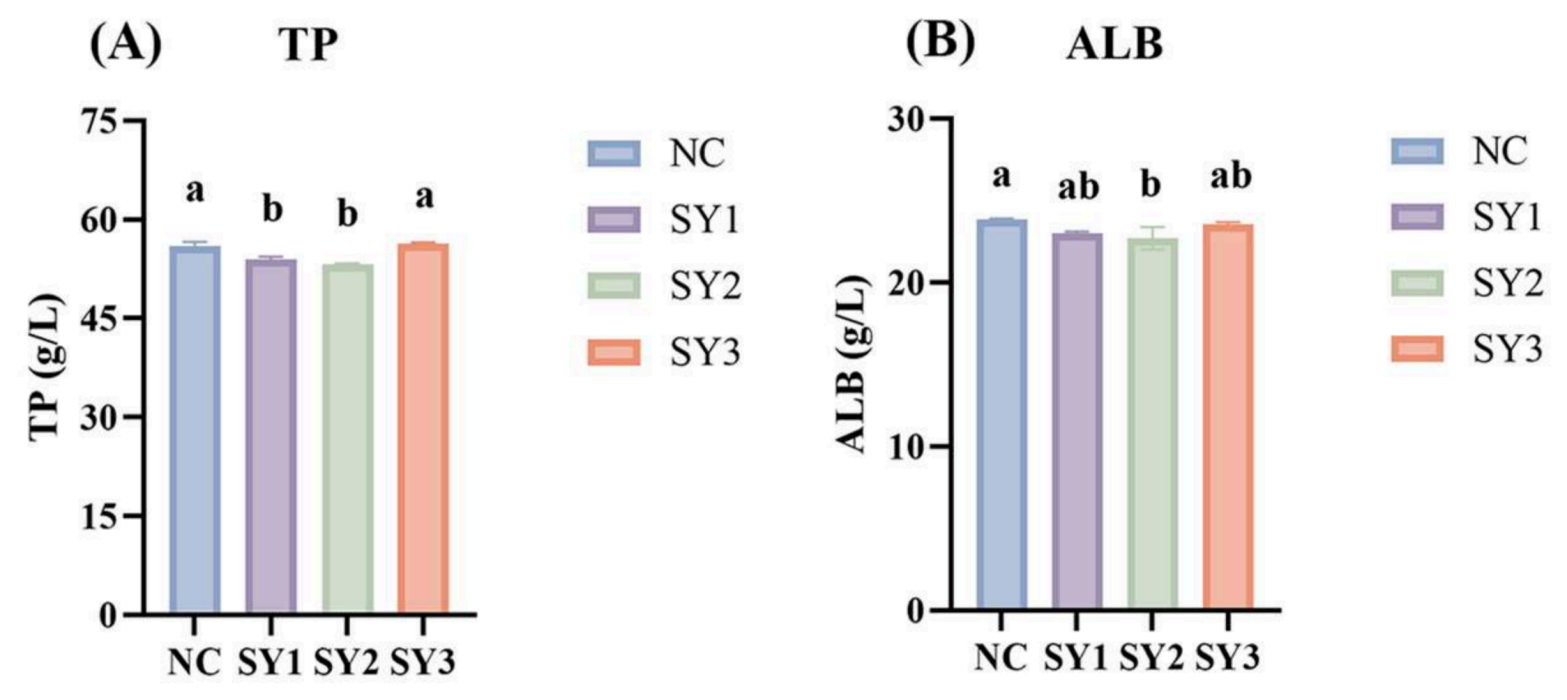
Effects of graded dietary SY supplementation on protein metabolism of laying hens. NC, negative control; SY1, 0.1 mg/kg; SY2, 0.3 mg/kg; SY3, 0.5 mg/kg. (**A**) TP, total protein. (**B**) ALB, albumin. The bars labeled a–b with no common superscript differ significantly (*p* < 0.05). *n* = 3 intra-cage sub-replicates per treatment (5 laying hens per sub-replicate, 15 hens total per group).

Protein metabolism parameters are illustrated in Figure 4. NC had a TP concentration of 55.93 ± 0.65 g/L. TP levels in SY1 and SY2 were significantly lower than those in NC (*p* < 0.001), and no significant difference was observed between SY1 and SY2 (*p* > 0.05). The minimum TP concentration (53.20 ± 0.17 g/L) was found in SY2, while TP in SY3 was similar to NC (*p* > 0.05). The ALB concentration of NC was 23.87 ± 0.06 g/L, and a significant reduction in ALB (22.70 ± 0.70 g/L) was only observed in SY2 (*p* = 0.004). ALB levels in other groups had no significant changes (*p* > 0.05).

#### 3.4.4. Serum Lipid Metabolism

**Figure 5:**
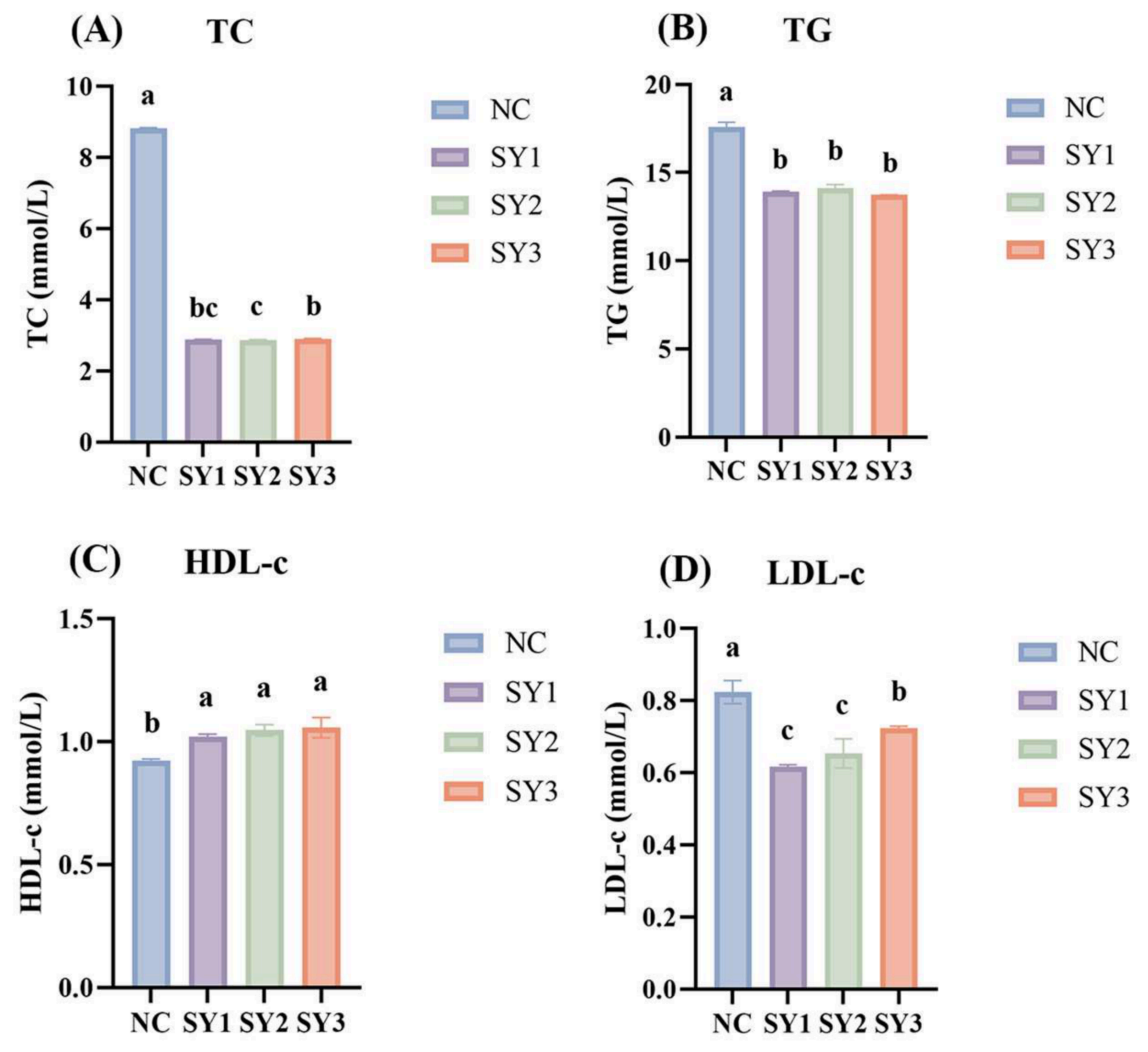
Effects of graded dietary SY supplementation on lipid metabolism of laying hens. NC, negative control; SY1, 0.1 mg/kg; SY2, 0.3 mg/kg; SY3, 0.5 mg/kg. (**A**) TC, total cholesterol. (**B**) TG, triglyceride. (**C**) HDL-c, high-density lipoprotein cholesterol. (**D**) LDL-c, low-density lipoprotein cholesterol. The bars labeled a–c with no common superscript differ significantly (*p* < 0.05). *n* = 3 intra-cage sub-replicates per treatment (5 laying hens per sub-replicate, 15 hens total per group).

Figure 5 shows lipid metabolism parameters of laying hens. NC had a TC level of 8.82 ± 0.02 mmol/L. TC concentrations in all SY groups were significantly lower than those in NC (*p* < 0.001), and a significant difference was detected between SY2 and SY3 (*p* = 0.009). SY2 had the lowest TC value at 2.86 ± 0.02 mmol/L. The TG concentration of NC was 17.60 ± 0.25 mmol/L. All SY groups had decreased TG levels (*p* < 0.001), and no significant difference was found among SY1, SY2, and SY3 (*p* > 0.05). The lowest TG concentration (13.73 ± 0.01 mmol/L) was recorded in SY3. The HDL-c level of NC was 0.92 ± 0.01 mmol/L. HDL-c concentrations were significantly increased in all SY groups (*p* = 0.001, *p* < 0.001, and *p* < 0.001, respectively), with no significant differences among SY treatments (*p* > 0.05). SY3 had the highest HDL-c concentration at 1.06 ± 0.04 mmol/L. The LDL-c concentration of NC was 0.82 ± 0.03 mmol/L. LDL-c concentrations in all SY groups were significantly reduced (*p* < 0.001, *p* < 0.001, and *p* = 0.002, respectively), and the minimum LDL-c value (0.62 ± 0.01 mmol/L) was observed in SY1.

#### 3.4.5. Humoral Immunity

**Figure 6:**
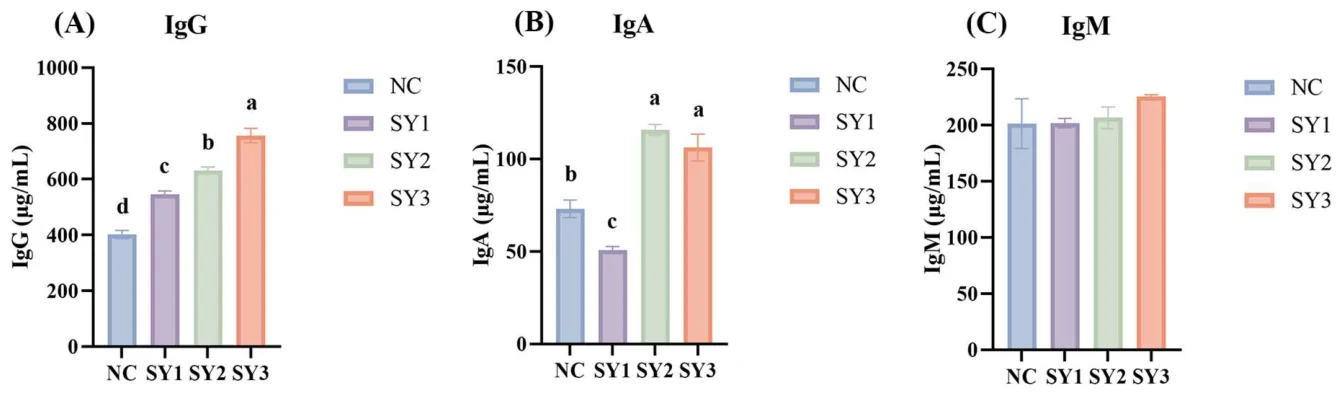
Effects of graded dietary SY supplementation on immune parameters of laying hens. NC, negative control; SY1, 0.1 mg/kg; SY2, 0.3 mg/kg; SY3, 0.5 mg/kg. (**A**) IgG, immunoglobulin G. (**B**) IgA, immunoglobulin A. (**C**) IgM, immunoglobulin M. The bars labeled a–d with no common superscript differ significantly (*p* < 0.05). *n* = 3 intra-cage sub-replicates per treatment (5 laying hens per sub-replicate, 15 hens total per group).

Humoral immune parameters are presented in Figure 6. IgG concentration increased linearly with the elevation of dietary SY dosage, and significant differences were found across NC, SY1, SY2, and SY3 (*p* < 0.001). SY3 had the highest IgG concentration at 756.30 ± 25.96 μg/mL. IgA levels in SY groups showed a trend of initial decline followed by a rise. IgA concentrations in SY2 and SY3 were significantly higher than those in NC (*p* < 0.001), and no significant difference existed between SY2 and SY3 (*p* > 0.05). The peak IgA level (115.80 ± 2.90 μg/mL) was detected in SY2. No significant difference in IgM concentration was observed between NC and all SY groups (*p* > 0.05).

### 3.5. Multivariate Comprehensive Analysis of Laying Performance, Egg Quality, and Serum Biochemical Parameters

To systematically clarify the internal correlation of multiple biochemical parameters and holistic physiological differences induced by graded SY supplementation, correlation heatmaps, hierarchical clustering, PCA, and a standardized radar chart were performed.

#### 3.5.1. Clustering and Correlation Analysis

**Figure 7:**
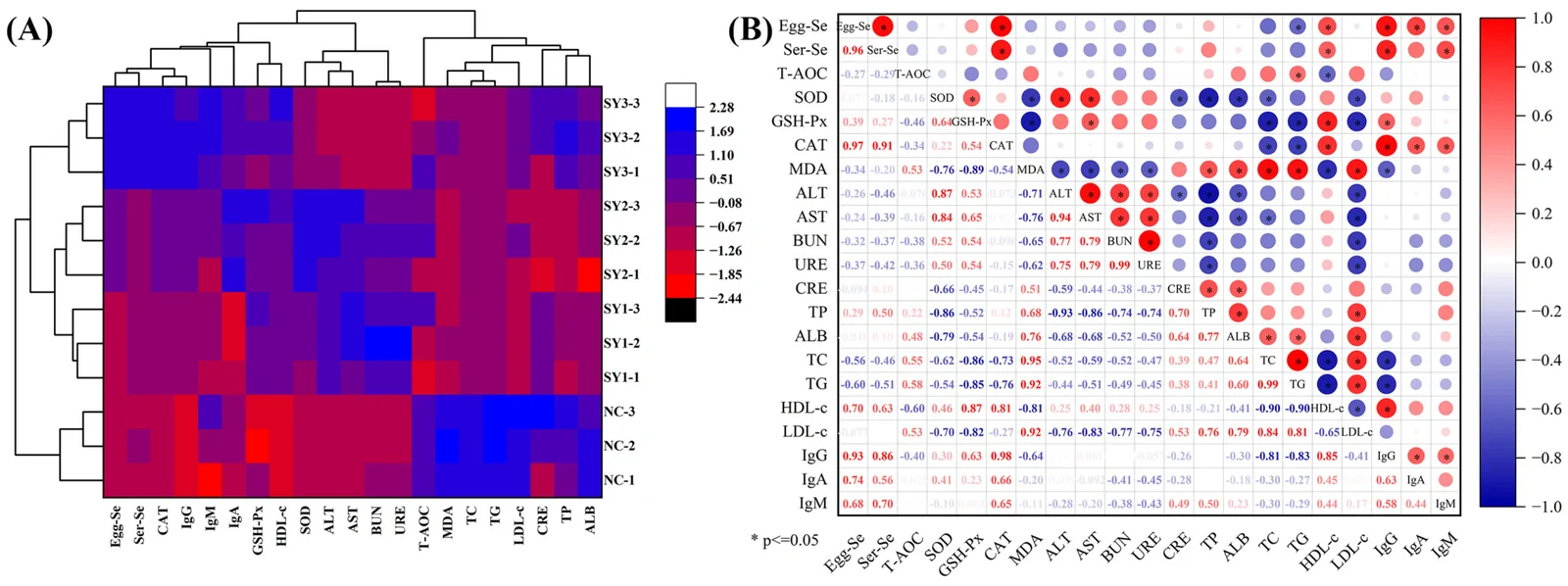
Clustering and correlation of serum biochemical parameters of laying hens fed graded SY. NC, negative control; SY1, 0.1 mg/kg; SY2, 0.3 mg/kg; SY3, 0.5 mg/kg. (**A**) Hierarchical clustering heatmap. (**B**) Pearson correlation heatmap. Color gradient represents normalized relative values: blue for low levels and red for high levels.

Hierarchical clustering heatmap of serum biochemical parameters across four treatment groups is shown in Figure 7A. Samples from the same group were clustered into independent branches, and four groups formed two major clusters: the NC group was separated from the three SY groups. The clustering profile further verified that graded SY supplementation remodeled the overall serum physiological characteristics of laying hens.

Figure 7B illustrates the pairwise correlation of all serum biochemical parameters. Egg-Se and Serum-Se exhibited strong positive correlations with all antioxidant parameters (T-AOC, SOD, GSH-Px, CAT) and humoral immune parameters (IgG, IgA, IgM). By contrast, the lipid peroxidation marker MDA and serum lipid metabolites (TC, TG, LDL-c) showed prominent negative correlations with Egg-Se and Serum-Se. Notably, hepatic and renal functional biomarkers (ALT, AST, BUN, URE) were moderately positively correlated with both Egg-Se and Serum-Se.

#### 3.5.2. PCA of Serum Biochemical Parameters

**Figure 8:**
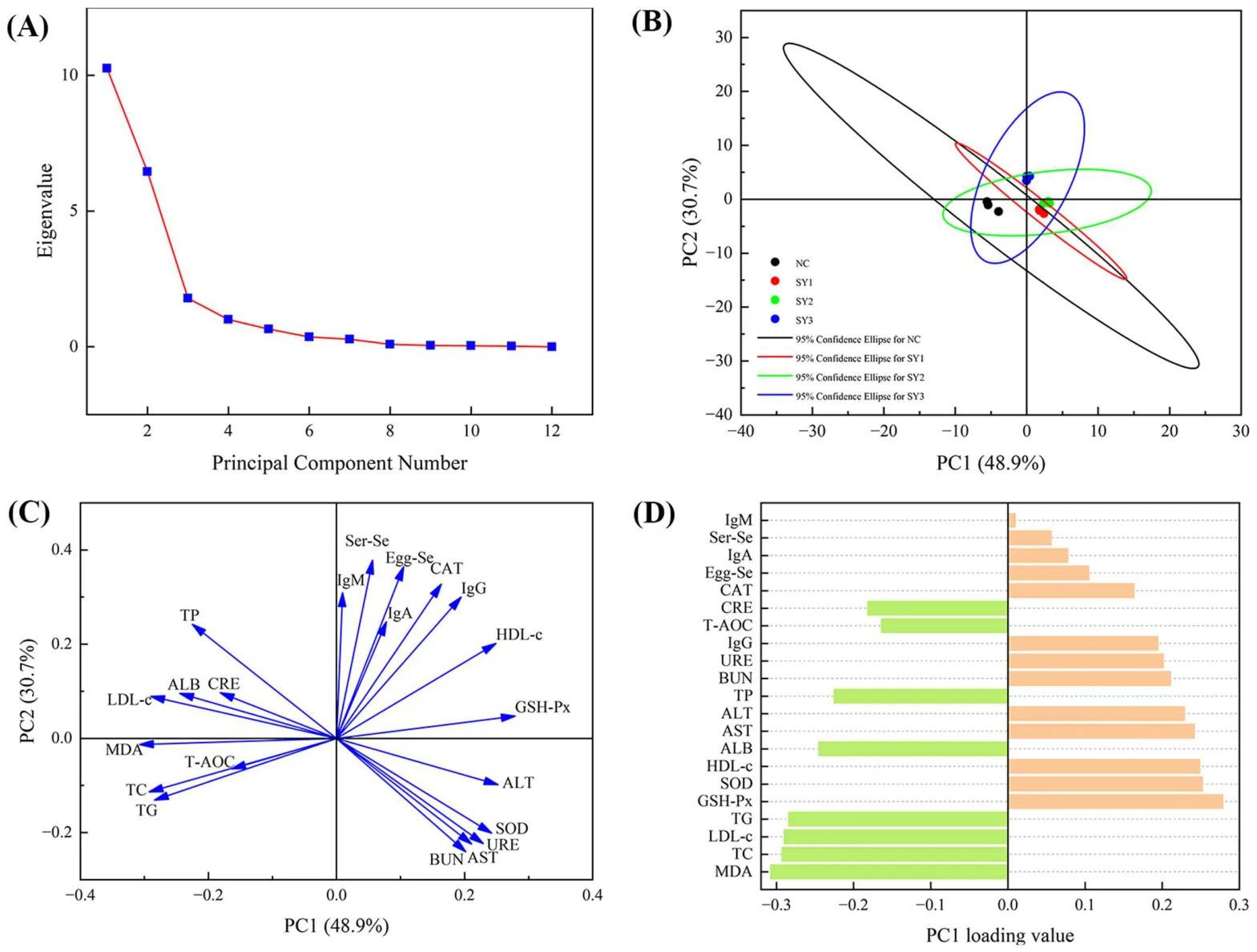
PCA of serum biochemical parameters of laying hens receiving graded SY. NC, negative control; SY1, 0.1 mg/kg; SY2, 0.3 mg/kg; SY3, 0.5 mg/kg. (**A**) Scree plot displaying eigenvalues of all principal components. (**B**) PCA score plot with 95% confidence ellipses. (**C**) PCA biplot integrating sample scatter points and variable loading vectors of all serum biochemical parameters. (**D**) Horizontal bar chart sorted by absolute PC1 loading values. Orange bars indicate biomarkers with positive PC1 loading values; green bars indicate biomarkers with negative PC1 loading values.

PCA was further applied to quantify the overall intergroup divergence and screen core differential serum biomarkers. The scree plot of eigenvalues is shown in Figure 8A. The first two principal components (PC1 and PC2) exhibited obviously higher eigenvalues than subsequent components, which together explained 79.6% of the total variance (PC1 = 48.9%, PC2 = 30.7%). Therefore, PC1 and PC2 were selected for subsequent dimensionality reduction and visualization.

The PCA score plot with 95% confidence ellipses for four treatment groups is presented in Figure 8B. Samples within each group were tightly aggregated, and the 95% confidence ellipse of the NC control group was completely separated from the ellipses of SY2 and SY3 high-dose Se groups. The SY1 group showed partial overlap with other Se supplementation groups, indicating that low-dose SY only induced mild alteration of serum biochemical parameters, while medium and high doses significantly reshaped the overall biochemical characteristics of laying hens.

PCA biplot, simultaneously displaying sample distribution and variable loading vectors, was illustrated in Figure 8C. Parameter vectors distributed along the positive PC1 axis mainly included antioxidant enzymes (GSH-Px, SOD, CAT), Se deposition markers (Egg-Se, Serum-Se), and immunoglobulins (IgG, IgA, IgM), which were enriched in SY2 and SY3 groups. By contrast, lipid metabolism parameters (TC, TG, LDL-c) and MDA were distributed along the negative PC1 axis, which were dominant in the NC group. Hepatic and renal function parameters were mainly distributed along the positive PC2 axis. Vectors with narrow angles represented strong positive correlations, while vectors with opposite directions represented negative correlations, which were consistent with the previous Pearson correlation heatmap results.

A horizontal bar chart sorted by absolute PC1 loading values was shown in Figure 8D to quantify the contribution of each index to the primary differential dimension PC1. MDA, TC, LDL-c, and TG exhibited the highest negative loading values, while GSH-Px, SOD, HDL-c, and IgG possessed the top positive loading values, which were identified as core differential biomarkers driving the separation between NC and SY groups.

#### 3.5.3. Comprehensive Physiological Characteristic Profiling

**Figure 9:**
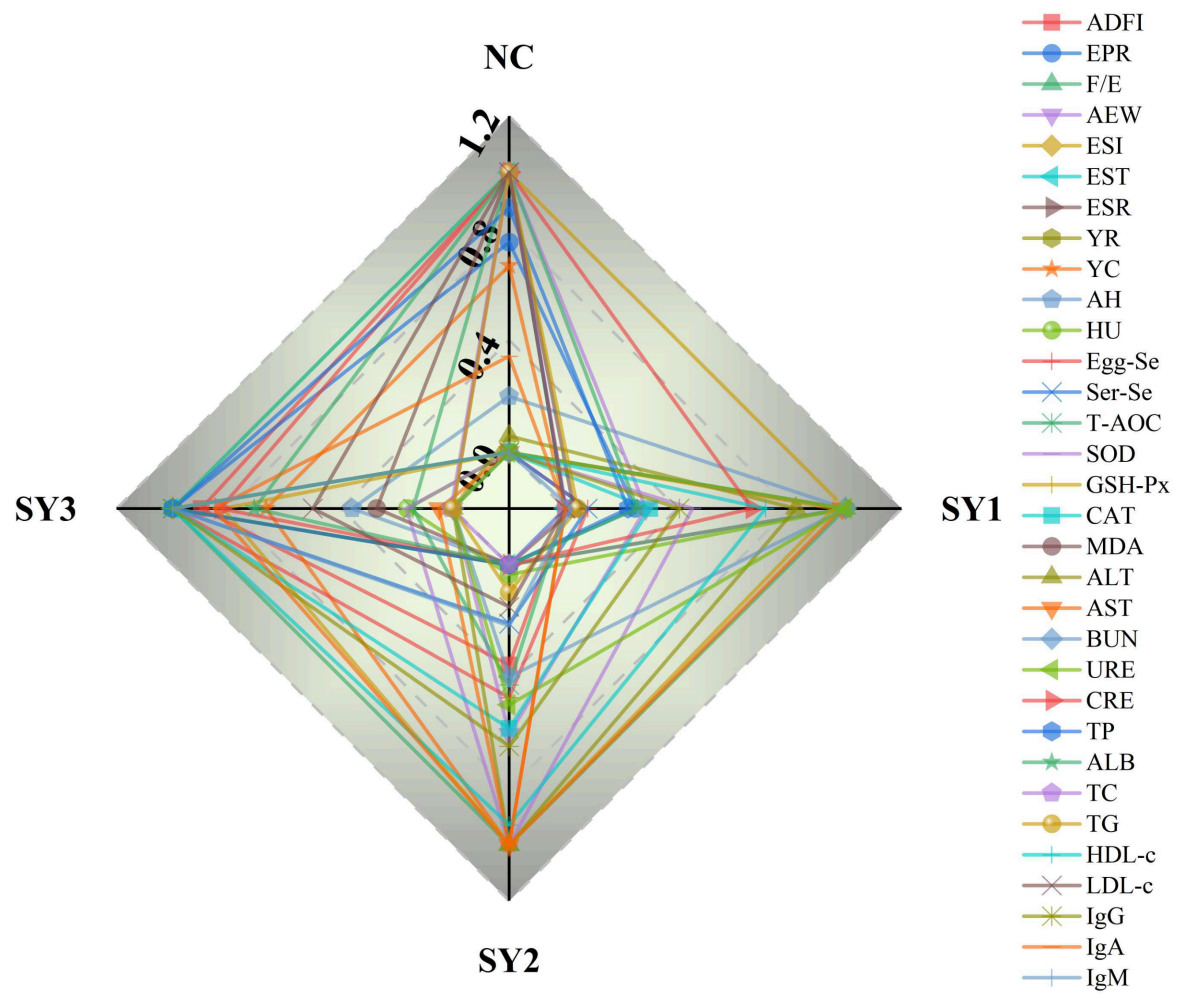
Standardized radar chart reflecting comprehensive physiological characteristic profiles of laying hens under different SY treatments. NC, negative control; SY1, 0.1 mg/kg; SY2, 0.3 mg/kg; SY3, 0.5 mg/kg. All detected parameters were normalized to a unified range of 0–1 to eliminate dimensional differences and each axis corresponds to one measured biochemical parameter.

A standardized radar chart covering all measured laying performance, egg quality, and serum biochemical parameters was plotted to intuitively compare the comprehensive physiological characteristics of the four treatment groups (Figure 9). All parameters were normalized to a uniform range of 0–1 to eliminate dimensional and magnitude differences.

No obvious inter-group divergence was observed on the axes of laying performance and egg quality, as these traits showed no significant differences across treatments. Consistent with PCA outcomes, the SY2 and SY3 groups displayed outward expansion along the axes of egg Se content, serum Se content, antioxidant enzymes, and immunoglobulins, representing stronger Se deposition, antioxidant capacity, and immune function relative to the NC and SY1 groups. The NC group exhibited prominent outward extension on MDA, TC, TG, and LDL-c axes, implying aggravated lipid peroxidation and dysregulated serum lipid metabolism under Se deficiency. The SY1 group presented an intermediate overall profile between the NC and medium/high-Se groups.

Collectively, the radar chart visually verified that graded SY supplementation coordinately improved Se accumulation, antioxidant capacity, and immune function, and alleviated Se-deficiency-induced lipid metabolic disorders. The physiological benefits reached a plateau at 0.3 mg/kg Se; no additional obvious advantages were observed when the dosage increased to 0.5 mg/kg Se.

## 4. Discussion

### 4.1. Laying Performance and Egg Quality

SY supplementation at 0.1–0.5 mg/kg Se did not alter laying performance and egg quality in this trial. Several similar findings indicate that within the low dosage range, SY exerts no influence on egg production, egg weight, feed conversion ratio, or internal egg quality, at least over a short-term feeding period [34,35,36]. In contrast, some studies have reported positive effects under different conditions. For instance, improved egg production rate and feed-to-egg ratio were observed in Lohmann Brown hens or aged laying hens (76 weeks old) with 0.3 mg/kg Se from SY [7,8], and a higher dose of 2 mg/kg Se was also reported to increase egg production rate [3]. Regarding egg quality, significant improvements in albumen height, Haugh unit, and yolk color were observed only at higher doses (≥1.5 mg/kg Se) after longer feeding periods (≥12 weeks) [2,17]. A meta-analysis by Yano et al. [27] confirmed that the effect of dietary Se on laying performance or egg quality is positive but highly variable, depending on baseline Se status, hen age, and strain. Therefore, improving egg quality usually requires higher Se doses or longer experimental cycles.

### 4.2. Se Deposition in Eggs and Serum

In this trial, egg and serum Se concentrations rose in a significant dose-dependent manner as dietary SY increased from 0 to 0.5 mg/kg. Such dose-responsive Se accumulation has been well documented in numerous prior layer studies [2,9,16,17]. Unlike prior layer trials, we developed a quadratic predictive model with high goodness of fit specifically for Hy-Line Brown hens (Figure 1B). This model quantifies the link between dietary Se supplementation and egg Se concentrations and further confirms the dose-dependent accumulation of organic Se in hen tissues. Existing literature has documented that SY delivers superior Se deposition efficiency, which can be attributed to its high proportion of selenomethionine. Selenomethionine is absorbed via intestinal amino acid transporters and directly incorporated into egg proteins [2].

### 4.3. Antioxidant Capacity

GSH-Px is a Se-dependent selenoprotein that serves as a core antioxidant barrier. SOD and CAT coordinate to eliminate reactive oxygen. T-AOC mirrors the comprehensive antioxidant reserve of serum, while MDA acts as a marker for lipid peroxidation injury [1,34]. Combined detection of this panel of parameters can comprehensively evaluate the antioxidant status in poultry.

Li et al. [14] verified that dietary SY supplementation significantly increased serum GSH-Px and SOD activities and reduced MDA accumulation. Meng et al. [37] reported that Se supplementation elevated serum T-AOC, CAT, and SOD activities, even though neither enzyme relies on Se as a cofactor. The favorable effects of Se on antioxidant defense systems are attributed to its role in selenocysteine synthesis. Specifically, Selenocysteine acts as a core component of GSH-Px to decompose harmful peroxides and alleviate oxidative damage [38,39]. Consistent with the above findings, our research found that the 0.3 mg/kg Se treatment exerted the optimal comprehensive antioxidant effect, accompanied by peak antioxidant enzyme activities (SOD and GSH-Px) and the lowest MDA level. When dietary supplemental Se was raised to 0.5 mg/kg, only CAT activity was further elevated, while SOD activity decreased and no additional antioxidant benefits were observed. Similarly, Qin et al. [40] reported that the antioxidant response seemed to plateau and even begin to decline as the SY level increased to 0.45 mg/kg. Although increases in SY may enhance antioxidant capacity within a certain range, surpassing an optimal threshold may likely trigger metabolic adaptation or regulatory mechanisms that maintain the antioxidant system at a physiological maximum. Consequently, further increases in SY dosage may not confer additional antioxidant benefits. Nrf2, a transcription factor critical for cellular protection against oxidative stress, is regulated by Keap1, whose reduced expression may promote Nrf2 activation and downstream antioxidant responses [41]. Fan et al. [16] demonstrated that dietary supplementation of Se-enriched *Lactobacillus plantarum* (0.3–0.4 mg/kg) offers a potent nutritional strategy to delay reproductive senescence by reinforcing the Nrf2-mediated antioxidant shield. These results support the hypothesis that SY modulates liver antioxidant defenses by influencing Nrf2 signaling pathways.

However, conflicting results have been reported in published research. Delezie et al. [42] found no significant shifts in serum GSH-Px activity after adding 0.1–0.5 mg/kg organic Se to laying hen diets. Chen et al. [3] demonstrated that ultra-high SY supplementation (2 mg/kg) only increased SOD activity, with no remarkable changes in T-AOC or GSH-Px levels. Variations may stem from differences in Se sources, bioavailability, and dosages.

### 4.4. Lipid Metabolism

TC, TG, and LDL-c are core serum lipid markers reflecting systemic lipid deposition and peroxidation damage, while HDL-c is a protective lipoprotein responsible for reverse TC transport and alleviating lipotoxic injury [5,43]. Combined detection of the above lipid parameters can provide a comprehensive assessment of the regulatory effect of dietary SY on serum lipid homeostasis in laying hens.

Wang et al. [4] supplemented graded organic Se, including SY, to diets of Lohmann Brown laying hens, and confirmed that all groups receiving 0.3 mg/kg Se had significantly lower serum TC and TG relative to the Se-free control. Ibrahim et al. [44] detected simultaneous reductions in plasma TC, TG, and LDL-c alongside increased HDL-c by feeding growing female turkey diets containing 0.4 mg/kg Se nanospheres. In line with the above studies, our study illustrated that proper SY supplementation positively regulates TC, TG, LDL-c, and HDL-c. This may be related to Se involvement in GSH-Px synthesis. GSH-Px regulates lipid metabolism by scavenging lipid peroxidation products in poultry.

Nevertheless, several inconsistent outcomes have been reported. Muhammad et al. [8] supplemented 0.15–0.45 mg/kg SY to 50-week-old Lohmann Brown laying hens, and found no statistically significant differences in serum TC, TG, HDL-c or LDL-c. Liu et al. [21] also reported negligible shifts in serum lipid parameters of aged laying hens receiving low-dose organic Se under non-stress feeding environments. Such discrepancies are mainly derived from variations in laying hen strain, growth stage, basal dietary composition, experimental duration, and breeding stress intensity.

### 4.5. Humoral Immunity

Humoral immunity constitutes a vital component of the body’s immune response, which exerts immune effects by blocking pathogen invasion and proliferation through antigen–antibody binding. IgA, IgG, and IgM are the primary effector molecules of humoral immune responses and can mirror the host’s humoral immune status [45].

One study reported that supplementation with 1.0 mg/kg Se from Se-enriched earthworm powder elevated serum albumin and IgG concentrations in laying hens [38]. Wang et al. observed that adding 0.3 mg/kg SY to the diets of 44-week-old Beijing Pink laying hens significantly increased egg IgG concentration. Dalia et al. [46] demonstrated that dietary organic Se derived from *Klebsiella pneumoniae* supplemented at 0.3 mg/kg markedly raised serum IgA and IgG levels in laying hens, without significant changes in IgM. Consistently, supplementation with 0.3 mg/kg SY in the diets of 28-week-old Hy-Line Brown laying hens effectively elevated serum IgG and IgA concentrations, with no significant influence on serum IgM levels in the present study. The difference in sensitivity among immunoglobulins is mainly attributed to their distinct expression stages during immune responses [47]. Differences in the immunoglobulin types that respond to Se supplementation across existing studies are likely attributable to differences in Se sources and laying hen breeds adopted in respective trials.

### 4.6. Liver and Kidney Function and Protein Metabolism

AST and ALT serve as critical markers of liver function. BUN and CRE, products of purine and muscle metabolism, are primarily excreted by the kidneys and act as important parameters of renal function in poultry. Serum TP and ALB reflect systemic protein metabolism. The liver dominates protein synthesis, and the kidney excretes nitrogenous wastes, so combined detection of hepatic, renal, and serum protein parameters can reflect hens’ metabolic adaptation to SY.

Several published studies have recorded changes in hepatic enzyme parameters after Se supplementation. Zhang et al. [2] found that ALT and AST slightly rose at the 0.3 mg/kg supplemental level, while sustained hepatic biochemical abnormalities coupled with hepatocyte lesions only appeared at the ultra-high 6.0 mg/kg SY group. Qiu et al. [26] also recorded mildly elevated ALT and AST in Hy-Line Brown hens fed 0.1–0.4 mg/kg SY. Consistent with the changing trend of liver enzymes observed in the above research, the present study found that 0.1 and 0.3 mg/kg SY significantly increased serum ALT, AST, URE, and BUN while decreasing TP and ALB levels, and these parameters were close to the control group at the 0.5 mg/kg SY treatment. Zhou et al. [17] recorded that Se from SY exists as selenomethionine and is metabolized in the liver for selenoprotein synthesis, which may alter the allocation of hepatic amino acids and further affect circulating levels of liver enzymes and serum protein.

Several opposite hepatic enzyme responses and inconsistent serum protein results have been reported. Liu et al. [21] observed reduced serum AST and ALT in aged laying hens supplemented with 0.3 mg/kg SY, which differed from the liver enzymes detected in the late-peak hens in this trial. For serum protein parameters, Chen et al. [35] and Zhang et al. [11] both reported no significant differences in TP and ALB under graded SY supplementation, which was inconsistent with the protein concentrations recorded in the current experiment. The divergent measurement results among different trials may be related to variations in hen physiological stages and basal diets. This study has limitations because liver and kidney histopathological observations were not performed in this trial. Therefore, we cannot confirm whether the numerical changes in serum biochemical parameters correspond to irreversible cellular or visceral lesions. In-depth research needs to be carried out in the future to explore the regulatory mechanisms.

### 4.7. Multivariate Statistical Analysis Supports Comprehensive Screening of Optimal Se Dosage

Most relevant studies in this field rely on univariate one-way ANOVA or simple pairwise correlations to describe changes in measured parameters. A small number of studies adopting multivariate approaches perform principal component analysis on partial parameters separately or restrict multivariate analysis to intestinal microbiota and transcriptomic datasets [2,7,27]. To the best of our knowledge, this is the first study to integrate four multivariate visualization approaches (Figure 7, Figure 8 and Figure 9) to systematically characterize physiological responses of late-peak laying hens to graded SY supplementation. This strategy establishes a comprehensive evaluation system for screening optimal Se dosage.

Hierarchical clustering (Figure 7A) clearly separated the NC group from all SY-supplemented groups. This grouping pattern confirms that dietary SY alters serum biochemical parameters of laying hens at a global level, rather than inducing random fluctuations in individual parameters. Pearson correlation heatmaps (Figure 7B) further reveal inherent associations among all serum parameters. Egg Se and serum Se concentrations were significantly positively correlated with antioxidant enzymes and immunoglobulins, while notable negative correlations were observed with MDA, TC, and LDL-c. These coordinated correlation features indicate that SY simultaneously regulates redox homeostasis, lipid metabolism, and humoral immunity, instead of acting on individual physiological pathways. PCA (Figure 8) shows that PC1 and PC2 collectively explained 79.6% of the total variance in serum biochemical parameters, capturing most physiological differences induced by SY dosages. The 95% confidence ellipse of the NC group was completely separated from those of SY2 and SY3, and an obvious deviation existed between the confidence ellipses of SY2 and SY3. Antioxidant enzymes and immunoglobulins exhibited positive loadings on PC1, representing favorable physiological traits; MDA, TC, TG, and LDL-c were distributed along the negative PC1 axis, corresponding to markers of oxidative and lipid damage. The standardized radar chart (Figure 9) provides intuitive visual evidence consistent with PCA outputs. The SY2 group displayed the maximum expansion along axes of egg Se, serum Se, antioxidant enzymes, and immunoglobulins, with the lowest levels of injury-related parameters. In comparison, only CAT activity and egg Se concentration increased in the SY3 group, whereas multiple core protective biomarkers declined relative to SY2, without further improvement in overall physiological status. Collectively, all multivariate plots exhibited consistent trends and provided multi-dimensional unified evidence supporting 0.3 mg/kg Se as the optimal supplemental level.

### 4.8. Implications and Recommendations

The Se enrichment efficiency, laying hen physiological health, and breeding economic benefits were integrated to evaluate dietary Se supplementation levels in this trial. For egg commercial compliance, the egg Se content of the NC group was 0.211 mg/kg. Eggs from the SY1 group only reached 0.236 mg/kg with marginal elevation, which could not produce high-value Se-enriched eggs. Eggs of the SY2 group contained 0.358 mg/kg Se, complying with GH/T 1135-2017 [24] for commercial circulation. Calculated based on the average egg weight of 61.5 g, each egg contained 22.017 μg Se, which meets the daily Se intake requirement for adults (20–25 μg) [48]. Whereas SY3 eggs reached 0.521 mg/kg Se, exceeding the standard upper limit of 0.50 mg/kg.

Daily gross profit was adopted as the primary indicator for quantifying the overall economic benefits of each group (Table 4). The SY used in this trial cost 240 CNY/kg. Supplementation of 0.05, 0.15, and 0.25 g SY per kilogram diet increased basal feed costs by 0.012, 0.036, and 0.060 CNY per kilogram, respectively. The economic benefit of NC was significantly lower than that of all SY treatment groups. Among the three SY groups, their daily gross profits were relatively comparable. SY2 yielded a moderate daily gross profit of 5,540 CNY based on a flock of 10,000 laying hens. Eggs from SY1 did not meet the Se content standard and thus could not be traded stably at a premium price. By contrast, eggs from SY3 exceeded the upper limit of Se content and were ineligible for formal commercial circulation. Only eggs produced by SY2 conformed to national standards and could be sold at the premium price of Se-enriched eggs for long-term commercial operation.

In terms of laying hen physiological performance, the SY2 group exhibited peak serum antioxidant enzyme activities and immunoglobulin concentrations, accompanied by significantly improved lipid metabolism. The SY3 group showed no additional physiological benefits, yet long-term feeding would lead to Se accumulation in vivo and raise the risk of chronic Se toxicity. Overall, SY1 had the lowest economic benefits but weak Se deposition efficiency and limited physiological regulation, resulting in low application value. SY3 brought substantially higher breeding costs, produced non-compliant eggs, and posed potential hazards to the health of laying hens.

Comprehensive evaluation of egg Se enrichment efficiency, laying hen physiological health, and economic benefits confirmed that 0.3 mg/kg SY-derived Se is the optimal supplemental Se level for Hy-Line Brown laying hens.

### 4.9. Limitations and Future Directions

This experiment has several limitations. Firstly, the 8-week feeding period is insufficient to characterize the long-term cumulative effects of SY; longer feeding trials are needed to monitor persistent physiological alterations. Secondly, the number of experimental replicates was limited, which may restrict the reliability of the results. Thirdly, only Hy-Line Brown laying hens were tested, which restricts the applicability of the optimized dosage to other laying hen breeds. Lastly, this study merely detected serum biochemical parameters. Liver and kidney histopathological sections, tissue Se contents, as well as selenoprotein gene expression profiles were not determined, restricting in-depth exploration of underlying molecular mechanisms. Future investigations are warranted to elucidate the molecular pathways through which graded SY supplementation modulates Se deposition and systemic metabolism in laying hens.

## 5. Conclusions

In conclusion, dietary supplementation with 0.1–0.5 mg/kg Se from SY did not adversely affect laying performance or egg quality in 28- to 36-week-old Hy-Line Brown laying hens, while dose-dependently increasing egg and serum Se contents. Multivariate physiological profiling further revealed that moderate SY supplementation coordinately enhanced antioxidant capacity, humoral immunity, and lipid metabolism. Integrating egg Se enrichment efficiency, laying hen physiological health, and economic benefits, 0.3 mg/kg Se from SY is recommended as the optimal dosage for the standardized production of qualified Se-enriched eggs. The 8-week trial duration and single-breed limitation warrant further long-term multi-breed investigations to confirm these findings and conduct in-depth research to clarify regulatory mechanisms.

## 6. Patents

A Chinese invention patent covering the SY feeding strategy for laying hens described herein has been filed with the China National Intellectual Property Administration. Application No.: 202610602966.5, Filing Date: 30 April 2026. No patent rights were granted at the time of manuscript submission.

## Figures and Tables

**Table 1:** Composition and nutrient levels of the basal diet.

Ingredient	Content (%)	Nutrients	Content (%)
Corn	61.0	Metabolizable Energy (MJ/kg)	11.2
Soybean meal	21.0	Moisture	10.0
Soybean oil	1.00	Crude Ash	12.5
Dicalcium phosphate	1.50	Crude Protein	14.9
Limestone	9.00	Crude Fat	3.81
Wheat bran	4.50	Crude Fiber	3.00
Premix ^1^	2.00	Total Phosphorus	0.557
Total	100	Available Phosphorus	0.325
		Calcium	3.83
		Methionine ^2^	0.373
		Lysine ^2^	0.882
		Nitrogen-Free Extract	55.8

^1^ The premix provides per kilogram of diet: Vitamin A 15,000 IU, Vitamin D_3_ 3000 IU, Vitamin E 30 mg, Vitamin K 2 mg, Vitamin B_1_ 2 mg, Vitamin B_2_ 4.5 mg, Vitamin B_6_ 2.2 mg, Vitamin B_12_ 0.04 mg, Niacin 30 mg, Pantothenic acid 6 mg, Folic acid 0.7 mg, Biotin 0.25 mg, Iron 70 mg, Copper 8 mg, Zinc 80 mg, Iodine 0.3 mg, Manganese 90 mg. ^2^ These values are measured values, other nutrients are calculated values.

**Table 2:** Effect of dietary SY on laying performance of hens.

Item	NC	SY1	SY2	SY3	*p*-Values
AEW, g	62.1 ± 0.9	60.9 ± 0.7	61.5 ± 1.0	60.5 ± 0.9	0.960
EPR, %	78.6 ± 9.0	77.1 ± 7.6	77.5 ± 5.3	80.0 ± 8.2	0.728
F/E	2.48 ± 0.07	2.55 ± 0.21	2.56 ± 0.19	2.63 ± 0.22	0.107
ADFI, g	122.5 ± 2.6	121.6 ± 2.8	121.9 ± 3.1	122.4 ± 3.2	0.749

Abbreviations: NC, negative control; SY1, 0.1 mg/kg; SY2, 0.3 mg/kg; SY3, 0.5 mg/kg; AEW, Average egg weight; EPR, Egg production rate; F/E, feed-to-egg mass ratio; ADFI, Average daily feed intake. Note: In the same row, values without letter superscripts indicate no significant difference (*p* > 0.05). *n* = 3 intra-cage sub-replicates per treatment (5 laying hens per sub-replicate, 15 hens total per group).

**Table 3:** Effect of dietary SY on egg quality of laying hens.

Item	NC	SY1	SY2	SY3	*p*-Values
4 weeks					
Egg shape index (ESI)	0.774 ± 0.026	0.767 ± 0.016	0.760 ± 0.026	0.768 ± 0.023	0.412
Eggshell thickness (EST), mm	0.410 ± 0.020	0.398 ± 0.021	0.402 ± 0.025	0.396 ± 0.024	0.397
Eggshell ratio (ESR)	0.128 ± 0.005	0.130 ± 0.016	0.128 ± 0.010	0.129 ± 0.008	0.818
Yolk ratio (YR)	0.265 ± 0.018	0.277 ± 0.017	0.275 ± 0.015	0.269 ± 0.018	0.222
Yolk color (YC)	5.40 ± 0.89	4.80 ± 0.45	5.60 ± 1.34	5.00 ± 0.71	0.508
Albumen height (AH), mm	5.06 ± 1.02	5.14 ± 0.75	5.18 ± 0.64	5.12 ± 0.90	0.997
Haugh unit (HU)	71.4 ± 6.3	69.9 ± 5.5	73.8 ± 4.3	72.1 ± 4.1	0.773
8 weeks					
Egg shape index (ESI)	0.772 ± 0.013 ^a^	0.773 ± 0.016 ^a^	0.759 ± 0.025 ^b^	0.769 ± 0.018 ^ab^	0.013
Eggshell thickness (EST), mm	0.430 ± 0.015	0.419 ± 0.019	0.418 ± 0.021	0.426 ± 0.015	0.237
Eggshell ratio (ESR)	0.120 ± 0.004	0.117 ± 0.005	0.119 ± 0.006	0.122 ± 0.005	0.606
Yolk ratio (YR)	0.283 ± 0.014	0.287 ± 0.018	0.280 ± 0.017	0.289 ± 0.018	0.178
Yolk color (YC)	6.00 ± 0.71	5.20 ± 0.45	6.40 ± 0.89	6.00 ± 0.71	0.094
Albumen height (AH), mm	6.17 ± 0.64	6.97 ± 0.54	5.97 ± 0.22	6.33 ± 0.57	0.126
Haugh unit (HU)	76.6 ± 5.4	82.9 ± 4.4	76.8 ± 0.7	77.6 ± 4.3	0.200

Abbreviations: NC, negative control; SY1, 0.1 mg/kg; SY2, 0.3 mg/kg; SY3, 0.5 mg/kg. Note: In the same row, values without letter superscripts indicate no significant difference (*p* > 0.05). The results labeled a,b with no common superscript differ significantly (*p* < 0.05). *n* = 3 intra-cage sub-replicates per treatment (5 laying hens per sub-replicate, 15 hens total per group).

**Table 4:** Economic benefits of laying hens fed diets supplemented with graded SY.

Item	NC	SY1	SY2	SY3
Egg market price, CNY/kg	10.0	20.0	20.0	20.0
Feed cost, CNY/kg	2.50	2.51	2.54	2.56
Total daily egg mass, kg	488	470	477	484
Total daily egg revenue, CNY	4880	9400	9533	9680
Total daily feed cost, CNY	3850	3901	3993	4073
Daily gross profit, CNY	1030	5499	5540	5607

Abbreviations: NC, negative control; SY1, 0.1 mg/kg; SY2, 0.3 mg/kg; SY3, 0.5 mg/kg. Note: All economic indicators were calculated based on 10,000 laying hens within a single-day accounting cycle. The laying performance parameters (AEW, EPR, F/E) required for calculating total daily egg mass and feed cost were sourced from Table 2.

## Data Availability

The original contributions presented in this study are included in the article. Further inquiries can be directed to the corresponding authors.

## Abbreviations

The following abbreviations are used in this manuscript:

Se	Selenium	AH	Albumen height
SY	Selenium-enriched yeast	YR	Yolk ratio
GSH-Px	Glutathione peroxidase	YC	Yolk color
Nrf2	Nuclear factor erythroid 2-related factor 2	EST	Eggshell thickness
SOD	Superoxide dismutase	T-AOC	Total antioxidant capacity
CAT	Catalase	MDA	Malondialdehyde
IgG	Immunoglobulin G	CRE	Creatinine
IgA	Immunoglobulin A	URE	Urea
ALT	Alanine transaminase	BUN	Blood Urea nitrogen
AST	Aspartate transaminase	TP	Total protein
NC	Negative control	ALB	Albumin
ADFI	Average daily feed intake	TC	Total cholesterol
EPR	Egg production rate	TG	Triglyceride
AEW	Average egg weight	HDL-c	High-density lipoprotein cholesterol
F/E	Feed-to-egg ratio	LDL-c	Low-density lipoprotein cholesterol
ESI	Egg shape index	IgM	Immunoglobulin M
ESR	Eggshell ratio	PCA	Principal Component Analysis
